# Chinese expert consensus on echelons treatment of thoracic injury in modern warfare

**DOI:** 10.1186/s40779-018-0181-6

**Published:** 2018-10-04

**Authors:** Zhao-Wen Zong, Zhi-Nong Wang, Si-Xu Chen, Hao Qin, Lian-Yang Zhang, Yue Shen, Lei Yang, Wen-Qiong Du, Can Chen, Xin Zhong, Lin Zhang, Jiang-Tao Huo, Li-Ping Kuai, Li-Xin Shu, Guo-Fu Du, Yu-Feng Zhao, Representing the Traumatology Branch of the China Medical Rescue Association, the Youth Committee on Traumatology Branch of the Chinese Medical Association, the PLA Professional Committee and the Youth Committee on Disaster Medicine, and the Disaster Medicine Branch of the Chongqing Association of Integrative Medicine, Quan-Wei Bao, Da-Qing Chen, Si-Xu Chen, Wei Cheng, Rui-Wu Dai, Jin Deng, Zai-Liang Ding, Tai-Qian Gong, Wei Guo, Shuai Hao, Ping Hu, Fei Huang, Gang Huang, Wei-Dong Jia, Shen Jiang, Hong-Xu Jin, De-Wen Kong, Xiang-Min Li, Xiao-Long Li, Lei Peng, Hao Qin, Bo Qu, Guo-hui Ren, Yin Shen, Hua Tang, Chen-chao Wang, Cheng Wang, Zhi-nong Wang, Wei Wu, Xu Wu, Zhao-xiang Wu, Wang Xi, Jian Xiao, Xiaokaiti Yibulayin, He-ping Xiang, Feng Xu, Peng Yan, Lei Yang, Jia-zhi Yang, Qian Yang, Yun-feng Yi, Chang-lin Yin, Yi-gang Yu, Rong Zhang, Song-qiang Zhang, Wan-sheng Zhang, Yu-feng Zhang, Yun-song Zhang, Xiao-gang Zhao, Yong-an Zhou, Zhao-wen Zong, Chang-ju Zhu

**Affiliations:** 1 State Key Laboratory of Trauma, Burn and Combined Injury, Department of War Wound Rescue Skills Training, Base of Army Health Service Training, Army Medical University, Chongqing, 400038 China; 2 Department of Cardiothoracic Surgery, Changzheng Hospital, Naval Medical University, Shanghai, 200003 China; 3 Department of Trauma Surgery, Daping Hospital, Army Medical University, Chongqing, 400042 China; 4 Special Clinic Department of Bethune Medical Profession Sergeant School, Shijiazhuang, 050000 China; 5 Institute of Health Service and Medical Information, Academy of Military Medical Sciences of the Chinese PLA, Beijing, 100850 China; 6 Department of Pharmacy, Naval Medical University, Shanghai, 200433 China

**Keywords:** Thoracic injury, Combat injuries, Echelons treatment, Expert consensus

## Abstract

The emergency treatment of thoracic injuries varies of general conditions and modern warfare. However, there are no unified battlefield treatment guidelines for thoracic injuries in the Chinese People’s Liberation Army (PLA). An expert consensus has been reached based on the epidemiology of thoracic injuries and the concept of battlefield treatment combined with the existing levels of military medical care in modern warfare. Since there are no differences in the specialized treatment for thoracic injuries between general conditions and modern warfare, first aid, emergency treatment, and early treatment of thoracic injuries are introduced separately in three levels in this consensus. At Level I facilities, tension pneumothorax and open pneumothorax are recommended for initial assessment during the first aid stage. Re-evaluation and further treatment for hemothorax, flail chest, and pericardial tamponade are recommended at Level II facilities. At Level III facilities, simple surgical operations such as emergency thoracotomy and debridement surgery for open pneumothorax are recommended. The grading standard for evidence evaluation and recommendation was used to reach this expert consensus.

In previous wars, thoracic injury accounted for approximately 4.4–33.0% of all wartime injuries. It is a major cause of injury and death in warfare and accounts for approximately 1–3% of all preventable wartime casualties [1–7]. In the United States (US)-led Operation Iraqi Freedom (OIF) and Operation Enduring Freedom (OEF), the epidemiological features and treatment needs for wartime thoracic injury were different than those in previous wars [3–6]. Currently, there are no uniform guidelines for the treatment of wartime thoracic injuries in the modern battlefield of the Chinese People’s Liberation Army (PLA). Therefore, we organized experts from the Chinese PLA Thoracic and Cardiovascular Surgery Professional Committee, the Army Medical University, the Naval Medical University, and the Academy of Military Medical Sciences to compile the “Chinese expert consensus on echelons treatment of thoracic injury in modern warfare” to provide a reference for the treatment of wartime thoracic injuries in the Chinese PLA.

Since the specialized treatment for wartime thoracic injury is basically the same as that for general conditions, only the guidelines of specialized treatment for wartime thoracic injury are introduced in this consensus. The guidelines contain three levels: first aid in the battlefield, emergency treatment, and early treatment, which are based on the currently used step care model in the Chinese PLA. The evidence and recommendation grades adopted in this expert consensus are mainly based on the standards recommended by the Oxford Evidence-Based Medicine Center and on the criteria commonly used in clinical studies, as we have previously described [8].

## Characteristics of epidemiological changes in thoracic injury in modern warfare

The available data show that the incidence of thoracic injury in previous wars was 4.4–33.0%. During the recent OEF/OIF, the incidence of thoracic injury was 8.6–10.5%, which was lower than that in World War II. This is mainly attributed to the widespread use of body armor, which effectively reduces the incidence of thoracic injury [1–7].

The mortality of thoracic injury has a different trend from its incidence. From the American Civil War to the Vietnam War, the mortality of thoracic injury has continued to decline. The mortality of wartime thoracic injury during the American Civil War, World War I, World War II, the Korean War, and the Vietnam War was 62.6%, 27.0%, 11.0%, 1.5%, and 2.9%, respectively [1–7]. Interestingly, during OEF/OIF, the mortality of thoracic injury “unexpectedly” increased to 10.5%. This was mainly due to the widespread use of protective equipment, improvement of first aid in the battlefield, rapid evacuation, which allows more soldiers with severe thoracic injuries to be evacuated to treatment facilities than in previous wars, and an improved registration system for wounded soldiers. All these improvements led to more soldiers with severe thoracic injuries being included in the statistics than those for previous wars, which led to the “unexpected” increase in the mortality of wounded soldiers with wartime thoracic injury in OEF/OIF [3–6]. Moreover, unlike previous wars such as the Vietnam War, blast injuries surpassed gunshot injuries in OEF/OIF and became a major cause of thoracic injury. A blast injury increases the incidence of visceral injury after blunt thoracic trauma and is difficult to diagnose and treat [9–12]. These specific changes require corresponding changes in treatment; for instance, intensive care for soldiers with severe thoracic injury is needed to reduce the mortality.

### Consensus 1

Due to changes in the injury mechanisms, the widespread use of protective equipment, and the improvement of first aid on the battlefield, thoracic injury in modern warfare is associated with an increased incidence of severe injury and organ injury caused by blunt trauma and with high mortality, which are different from the characteristics of thoracic injury observed in previous wars. Therefore, these changes require corresponding changes in treatment (Class B/Category I).

## Protective equipment can reduce the incidence of thoracic injury

As mentioned above, there has been a downward trend in the overall incidence of thoracic injury for US forces throughout all previous wars. This change is mainly attributed to the widespread use of body armor by US forces, which increases the protection of the torso [1]. Data from OIF/OEF show that effective protection significantly reduces the incidence and overall mortality of thoracic injury in US forces and their allies [2, 3].

### Consensus 2

Body armor and other protective equipment can effectively reduce the incidence, severity, and overall mortality of thoracic injury (Class B/Category I).

## First aid on the battlefield for current wartime thoracic injury

Tension pneumothorax accounted for 3–5% of all preventable wartime injuries during the Vietnam War [13]. In OIF and OEF, tension pneumothorax and open pneumothorax are the third cause of preventable wartime casualties, as timely and effective treatment can save over 90% of the wounded soldiers [14]. Therefore, it is necessary to differentiate tension pneumothorax from open pneumothorax without an auxiliary examination at the scene of the battlefield and to start emergency treatment.

In the battlefield, the following signs can be used to identify soldiers with tension pneumothorax: 1) history of chest injury; 2) progressive difficulty breathing, i.e., fast breathing and labored respiration; 3) attenuated or absent breath sound on the side of the injury; 4) elevated chest wall on the side of the injury compared with the contralateral side, subcutaneous emphysema, and jugular vein distension; and 5) hypotension and shock induced by tachycardia and shortness of breath, which can be worsened by the increased intrathoracic pressure. Chen et al. [13] analyzed the symptoms and signs of 111 cases of tension pneumothorax in the trauma database of the Israel Defense Forces from 2007 to 2012 and found that the most common clinical manifestations were attenuated breath sound on the side of the injury and shortness of breath. Loss of consciousness and absence of radial artery pulse are also common symptoms and are associated with high mortality. No tracheal deviation was observed in any cases in the database with tension pneumothorax. Moreover, it is very difficult to identify attenuated breath sound through a physical examination on the battlefield due to environmental noise. In OIF and OEF, US forces discovered that hand-held, miniaturized B-mode ultrasound instruments were helpful in the diagnosis of tension pneumothorax in the battlefield, and medics were able to use this technique after a short training session. Studies have shown that the sensitivity and specificity of ultrasound in the diagnosis of tension pneumothorax is 92.0% and 99.4%, respectively, which are higher than those of X-ray and similar to those of computed tomography (CT) scan [15–17].

Needle thoracentesis is required for wounded soldiers diagnosed with tension pneumothorax. The Advanced Trauma Life Support (ATLS) recommends the use of a 5-cm-long puncture needle to perform thoracentesis for the treatment of tension pneumothorax at the second intercostal space in the midclavicular line. After comparative measurements and clinical observations, studies revealed that the thickness of the chest wall of individual US soldiers was often more than 5 cm at the second intercostal space. Therefore, a 3.25-in.-long (8.25 cm) puncture needle (No. 14) is recommended by the Committee for Tactical Emergency Casualty Care (C-TECC) [18, 19]. The recommended site for needle thoracentesis is still at the second intercostal space in the midclavicular line [13, 20, 21].

After needle thoracentesis, it is still controversial whether or not to make a valve in the drainage tube in the battlefield. Some researchers argue that the diameter of the drainage tube is much smaller than that of the airway. Air circulation through the drainage tube could not significantly affect respiration. Without a valve, the thoracentesis can still convert tension pneumothorax into open pneumothorax, which is less severe. We support the idea of using a glove to make a one-way valve to increase the effectiveness of decompression if the battlefield conditions allow, especially when the estimated evacuation time is long or the evacuation may be delayed. The drainage tube can be placed in the puncture site and flushed with saline every 2 hours to ensure its patency. If conditions allow, the drainage tube can be removed, and the soldier should be monitored closely. If signs of tension pneumothorax are present, needle thoracentesis should be repeated. If necessary, tube thoracostomy should be performed. A study by Chen et al. [13] showed that 32% of wounded soldiers undergoing needle thoracentesis underwent tube thoracotomy in battlefield hospitals.

The main signs for the diagnosis of open pneumothorax include the following: 1) history of thoracic injury and presence of chest wall wounds; 2) sucking or hissing sounds in the chest wall and foamed blood in the wound; 3) difficulty breathing; and 4) chest wall unable to rise normally during inhalation. In 2013, the US C-TECC updated the treatment guidelines for open pneumothorax. Once open pneumothorax is confirmed, a breathable chest pad must be used immediately to close the wound. If a breathable chest pad is not available, a conventional chest pad can be used to close the wound, and then the soldier should be monitored closely for symptoms of tension pneumothorax. If the wounded soldiers suffer from persistently progressive hypoxia, respiratory distress, or hypotension, the occurrence of tension pneumothorax should be considered and the chest pad should be removed or needle thoracentesis should be performed for decompression [22, 23].

### Consensus 3

Wounded soldiers with a history of thoracic injury may be diagnosed with tension pneumothorax when they have symptoms of progressive dyspnea and attenuated breath sound on the side of the injury. Portable B-mode ultrasound instruments may help with the diagnosis when effective physical examinations cannot be performed on the battlefield due to environmental noise (Class B/Category IIb).

### Consensus 4

Once tension pneumothorax is confirmed, it is recommended to use a No. 14 puncture needle (8.25 cm in length) for needle thoracentesis at the second intercostal space in the midclavicular line. If conditions allow, a valve can be added at the end of the puncture needle. After needle thoracentesis, the wounded soldier should be closely monitored. When the symptoms of tension pneumothorax reoccur, needle thoracentesis should be repeated, or tube thoracostomy should be performed (Class B/Category IIa).

### Consensus 5

Wounded soldiers with a history of thoracic injury may be diagnosed with open pneumothorax if they have progressive dyspnea, sucking or hissing sounds in the chest wall, and foamed blood in the wound (Class B/Category IIb).

### Consensus 6

Once open pneumothorax is confirmed, a breathable chest pad can be used immediately to close the wound. If a breathable chest pad is not available, a conventional chest pad can be used to close the wound. The soldier should then be closely monitored for symptoms of tension pneumothorax. If the wounded soldiers suffer from persistently progressive hypoxia, respiratory distress, or hypotension, the occurrence of tension pneumothorax should be considered, and the chest pad should be removed or needle thoracentesis should be performed for decompression (Class B/Category IIa).

## Emergency treatment for wartime thoracic injury in modern warfare

During the level of emergency treatment, tension pneumothorax, open pneumothorax, massive hemothorax, flail chest, and pericardial tamponade need to be further observed and treated, and painkillers and tetanus antitoxin may be used.

### Identification and treatment of massive hemothorax

Chest pain and shortness of breath are two major symptoms of massive hemothorax, and some of the wounded soldiers may be accompanied by shock. Attenuated or absent breath sound can be noted on the side of the injury, and percussion dullness can be noted. In emergency treatment facilities, B-mode ultrasound instruments can be used in the diagnosis of hemothorax. When soldiers with thoracic injury suffer shortness of breath and no remission after needle thoracentesis, massive hemothorax should be considered. For soldiers considered to have massive hemothorax, tube thoracostomy should be performed, and in general, the drainage tube should be placed in the fourth/fifth intercostal space in the midaxillary line [24].

### Identification and emergency treatment of flail chest

In the emergency facilities of the Chinese PLA, X-ray and CT scan are not available, therefore the diagnosis of flail chest depends mainly on the clinical manifestations and signs. For soldiers with a thoracic injury with multiple rib fractures, rapid breathing, and shock, flail chest combined with lung injury (mainly pulmonary contusion) should be considered first. The presence of paradoxical movement of the chest wall is of great significance in the diagnosis of flail chest.

In emergency treatment facilities, paradoxical movement of the chest wall should be controlled as soon as possible, airway patency and adequate oxygen supply should be maintained, respiratory and circulatory dysfunction should be corrected, and shock should be prevented. When the softening chest wall is limited or located on the back, a local pad can be used as a pressure dressing. A paradoxical movement of the chest wall of 3–5 cm can cause severe respiratory and circulatory disorders and can quickly lead to death. Thus, emergency treatment must be applied. A pressure dressing with pads should be temporarily applied, and then the chest is fixed with a multi-head chest strap.

For soldiers with flail chest and pulmonary contusion, adequate tissue perfusion should be ensured without limitation. Once wounded soldiers are fully resuscitated, unnecessary fluid should be avoided. The appropriate methods should be selected to control the pain of the wounded soldiers to reduce the possibility of respiratory failure [25].

### Identification and emergency treatment of pericardial tamponade

For soldiers with penetrating chest injuries, medics should be aware of the possibility of pericardial tamponade. Blunt trauma located in the area, which is bordered by the horizontal line at the level of the clavicle, the vertical lines to the costal margin via the bilateral nipples, and the connecting line between the crossing points of the bilateral vertical lines to the costal margin line, may cause pericardial tamponade. The presence of Beck’s triad (distant and muffled heart sounds, distended jugular vein, and low arterial blood pressure) is the indication of pericardial tamponade in wounded soldiers. However, it is extremely difficult to find these phenomena in emergency treatment facilities, especially the muffled heart sounds. Therefore, medics in the battlefield should pay attention to the location of the injury and the sign of low blood pressure and give wounded soldiers the appropriate treatment. In this case, the use of B-mode ultrasound instruments can effectively improve the success rate of the diagnosis of pericardial tamponade, whereas electrocardiography (ECG) examination can only show low QRS voltage [26, 27].

Pericardiocentesis is an effective and common treatment for pericardial tamponade that works by draining fluid from the pericardial sac. Two specific puncture locations are described as follows: 1) Puncture under the xiphoid process, at the junction of the xiphoid process and the left costal margin. The puncture needle can be advanced toward the left side into the posterior-inferior part of the pericardial cavity at a 30–45° angle to the abdominal wall. 2) Puncture in the apex of the heart, with the puncture site located 2 cm within the border of cardiac dullness in the left fifth intercostal space or the sixth intercostal space. The needle is inserted at the upper edge of the rib and advanced slightly toward the midline into the pericardial cavity. Ultrasound can be used to guide the pericardiocentesis to reduce complications when the conditions allow. Wounded soldiers undergoing pericardiocentesis have priority to be evacuated to an early care facility for further assessment of injuries and auxiliary examinations, in order to clarify the condition of the heart injury and receive effective treatment.

### Further observation and treatment of pneumothorax

Tube thoracostomy should be performed if the symptoms are not obviously relieved or even progressively worsening in soldiers with tension pneumothorax undergoing needle thoracentesis in the battlefield. If there is no accompanying hemothorax, the drainage tube can be inserted into the second and third intercostal space. It should be noted that wounded soldiers with a drainage tube are still at great risk to develop tension pneumothorax during evacuation, especially if they are under positive pressure ventilation. If signs of tension pneumothorax begin to appear, kinking in the chest tube or connecting tube should be excluded first; then, the connection from the connecting tube to the liquid seal and drainage equipment should be secured. Even if there are no severe symptoms of tension pneumothorax, soldiers who have gradually developed symptoms of tension pneumothorax may need needle thoracentesis.

### Consensus 7

When symptoms such as chest pain, shortness of breath, signs of shock, and attenuated breath sound on the side of the injury appear in wounded soldiers with a history of thoracic injury, massive hemothorax should be considered. In this case, it is recommended to insert the drainage tube in the fourth/fifth intercostal space for closed thoracic drainage (Class B/Category IIa).

### Consensus 8

If the symptoms of rapid breathing and shock appear in soldiers with thoracic injury and multiple rib fractures, flail chest accompanied by pulmonary contusion should be considered first. In emergency treatment facilities, paradoxical movement of the chest wall should be controlled as soon as possible. The patency of the airway should be maintained and tissue perfusion should be ensured under limited fluid resuscitation. Pain should also be controlled (Class A/Category I).

### Consensus 9

Wartime blunt and penetrating injury may lead to pericardial tamponade, and adequate alertness is an important factor for its identification. Beck’s triad, low QRS voltage from the ECG examination, and echocardiography can be used to support the diagnosis of pericardial tamponade. Pericardiocentesis is required in wounded soldiers with possible pericardial tamponade. The pericardiocentesis should be performed at the site under the xiphoid process or the apex of the heart. Ultrasound can be used to guide the operation to improve safety (Class B/Category IIa).

## Early treatments for wartime thoracic injury in modern warfare

At present, relatively simple surgical procedures such as emergency thoracotomy, debridement, and suturing of open pneumothorax can be performed in early treatment facilities in the Chinese PLA. As mentioned above, during modern warfare, soldiers with thoracic injury who are evacuated to Level III treatment facilities are usually in critical condition. If they are not treated in time, mortality is extremely high. The US forces’ experience in OIF and OEF shows that penetrating cardiac injuries, thoracoabdominal injuries, and diaphragm ruptures can be treated by cardiac repairs, removal of injured lung tissue, hemostasis, and diaphragm repairs in Level III facilities to save more lives [28–30].

### Resuscitative emergency thoracotomy

Indications for resuscitative emergency thoracotomy include short-term (usually no more than 15 min) cardiac arrest or impending cardiac arrest caused by penetrating and blunt trauma. The success rate of resuscitation emergency thoracotomy is 7–21%. However, the success rate is generally higher for some soldiers, particularly those who lose their life signs for less than 45 min and those who are evacuated to Level III facilities alive but experience cardiac arrest later and were treated by closed chest cardiopulmonary resuscitation (CC-CPR) for less than 15 min.

Resuscitative emergency thoracotomy should be performed on the basis of effective blood transfusions, fluid infusions, and other anti-shock treatments. The left-sided incision or clamshell approach is usually used to open the chest. The pleura and pericardium are opened, the injured aorta is clamped, and intrathoracic CPR is performed. If the heart resuscitation is successful, emergency procedures should be prepared immediately, and the wounded soldier should be transferred to the operating room for further surgical treatment [31–34].

### Damage control thoracotomy

The indications for damage control thoracotomy (DCT) in the battlefield include the following: 1) Wounded soldiers with massive and progressive hemorrhages in the chest cavity, including soldiers who have not improved after anti-shock treatment or improved temporarily and then deteriorated rapidly, whose initial drainage volume after closed thoracic drainage is more than 1000 ml or the drainage volume exceeds 200 ml per hour for more than 3 h. Severe lung lacerations and large intrathoracic blood vessel ruptures are the major causes of intra-thoracic hemorrhage during wartime. 2) Wounded soldiers with severe heart contusion. 3) Wounded soldiers with severe tracheal and bronchial injuries [34–37].

After DCT is confirmed, the left-sided anterolateral thoracotomy approach is generally used as an initial incision to expose the pericardium, descending aorta, proximal left subclavian arteries, and left hilum. If necessary, the clamshell approach can be used to improve the exposure of the pericardium, heart, and anatomical structure of the thoracic inlet. The main drawback of this approach is the insufficient exposure of the esophagus and trachea. After weighing the advantages and disadvantages of different approaches, the anterolateral incision is the best for exposure and control of urgent, fatal injuries. The median sternotomy is the most practical incision that can reach the heart and great vessels. It is can also be used to access the hilar structure and a small part of the lungs. The use of this incision is very common, and it can be extended through the “roof window” to the neck and adjacent upper extremity. This window provides access to the subclavian artery and its branches (including the proximal vertebral artery, internal mammary artery, and axillary artery). However, the disadvantages of the median sternotomy are as follows: the access to the lungs is not optimal, especially to the left lower lobe, and it fails to expose the posterior mediastinum structure, especially the descending aorta; sternum saws or a Lebsche knife are required for sternotomy in an adult, but on the battlefield, these power tools may not be available. A combined thoracoabdominal incision is an option for obtaining access to the lower chest and upper abdomen, but it is rarely used for emergency treatment [38].

After full exposure of the chest, appropriate treatment is performed based on the findings (see below for details). In terms of uncontrollable bleeding, gauze packs are recommended to control bleeding [39], and wounded soldiers should be given priority to be evacuated to specialist hospitals for treatment.

### Diagnosis and early treatment of severe lung laceration

Both penetrating and blunt trauma can cause lung lacerations. A large lung laceration can cause difficulty breathing, cyanosis, and rapid pulse. If the blood loss is considerable, blood pressure may drop, and shock may even occur. Most soldiers with lung lacerations can be cured by closed thoracic drainage. For soldiers with no obvious improvement in dyspnea and progressive hemothorax after closed thoracic drainage, thoracotomy is needed to find the sites of hemorrhage or leakage in order to suture them. If the laceration is too severe to be repaired, lobectomy or segmentectomy may be an option. Pneumonectomy is the last option for soldiers with lung or hilar tissue damage. The mortality of soldiers with unilateral pneumonectomy is greater than 50%. Large air leaks in the trachea or severe pulmonary hemorrhage can be temporarily controlled by cutting the inferior pulmonary ligament, clamping the hilum, or twisting the lung by 180° around the hilum. During pneumonectomy, the hilum should be clamped slowly to allow the other lung to adapt. The amount of fluid during resuscitation should be minimized to avoid the occurrence of acute right heart failure [36, 37]. Suture and ligation of hilar vessels and bronchial tubes should be performed individually with the support of pleura or other flexible soft tissue such as the internal jugular muscle.

### Diagnosis and early treatment of severe tracheal and bronchial injuries

Both penetrating and blunt trauma can cause the rupture of the trachea and bronchus, which leads to pneumothorax, airway obstruction by hemorrhage, and pulmonary contusion, resulting in dyspnea. After the rupture of the trachea, the typical manifestations of tracheal injury, such as mediastinal emphysema and subcutaneous emphysema in the suprasternal fossa, may quickly occur and spread to the neck, face, and chest. X-ray should show a collection of gas along the anterior edge of the spine, followed by signs of severe gas accumulation in the mediastinum. If there are mediastinal pleura ruptures, signs of pneumothorax or hydropneumothorax in the chest should be found.

Conservative treatment can be used for small ruptures of the intrathoracic trachea and bronchi. For soldiers with a large rupture, if a tracheotomy and closed thoracic drainage do not alleviate the dyspnea, surgical repair should be performed in an early treatment facility. The advantages of surgical treatment include early pulmonary re-expansion, prevention of stricture of the injury site, clear exposure of the rupture sites, and simplicity in operation [40, 41]. A thoracic surgeon should be available for the repair of the damaged trachea and bronchi in battlefield hospitals; if one is not available, the wounded soldiers should be given priority to be evacuated with tracheal intubation and ventilatory support.

### Early treatment of heart injury

More than 80% of soldiers with penetrating heart injuries die at the time of injury. Early diagnosis and surgery are the key factors for survival for wounded soldiers who are still alive when arriving at battlefield hospitals. The diagnosis of penetrating heart injury is usually confirmed by reliable clinical symptoms and ultrasound examination of pericardial trauma. Chest radiography may help in the diagnosis of penetrating heart injury and the valuable signs include enlarged heart shadow, widened superior mediastinum, and gas accumulation in the pericardial sac. Approximately 30% cases of penetrating heart injury could be diagnosed by ECG with the presence of the following signs: low QRS voltage, ST-segment elevation, inverted T waves, etc. [42, 43].

The diagnosis of blunt heart injury is challenging. Some of the wounded soldiers may experience tachycardia, arrhythmia, or cardiogenic shock. Laboratory tests may reveal elevated troponin levels and an abnormal ECG. Sufficient awareness is the key to improving the success rate of the diagnosis. Trauma ultrasound examination, ECG, and measurement of troponin levels should be performed on suspected wounded soldiers. If these examinations are normal, no further investigation is required; if the ECG is abnormal or the troponin levels are elevated, the soldier should be closely monitored by ECG [44]. For soldiers with penetrating and blunt heart injuries, early diagnosis is required, and surgical repair may provide a certain chance of survival.

### Early treatment of injury of the thoracic great vessels

Soldiers with penetrating trauma of the thoracic great vessels often die at the scene on the battlefield and do not have the chance to be evacuated to an early treatment facility. However, soldiers with blunt trauma of the great vessels may suffer from pain in the retrosternal or scapular area. Since the all-layer aortic wall rupture is covered by the mediastinal pleura, a hematoma may form and extend along the gap of the loose tissue to the chest and neck, resulting in pleural effusion, thickening of the neck, pulsatile masses, or compression to the jugular veins and descending aorta. If the hematoma tension is too great and the mediastinal pleura suddenly ruptures, the soldier may die because of the delayed massive bleeding. Physical examination should reveal a rough, blowing, systolic murmur in the precordial area and the interscapular area. X-rays can show signs of widening of the mediastinum and blurring or irregular outline of the thoracic aorta. CT angiography can improve the success rate of the diagnosis of great vascular injury [45, 46]. However, CT scanners are not available in Level III treatment facilities in the Chinese PLA.

Soldiers with an injury of the great vessels who do not suffer massive hemorrhage or shock should be quickly evacuated for specialist treatment under close monitoring. In the event of a progressive chest hemorrhage, thoracotomy should be performed urgently to repair damaged blood vessels, or vascular bypass surgery should be performed. In OIF and OEF, US forces reported successful stenting of the thoracic vessel in battlefield hospitals after blunt trauma [47, 48], but we do not recommend performing this procedure in battlefield hospitals under the existing technical and equipment conditions of the Chinese PLA.

### Early treatment of esophageal injuries

The incidence of wartime esophageal injuries is low. This type of injury is a result of a penetrating injury caused by a gunshot wound. Therefore, penetrating injuries near the esophagus usually lead to esophageal injury. Severe posterior sternal and epigastric pain, dyspnea, and cyanosis, as well as mediastinal and subcutaneous emphysema, are common symptoms and signs. Severe infections may cause sepsis and even shock [49]. Subcutaneous emphysema in the mediastinum or neck, which can be revealed by chest radiographs, is important for the diagnosis of esophageal injury [49]. The US forces, which are equipped with esophagoscopes in battlefield hospitals and trained to perform an esophagoscopy, can make a much more accurate diagnosis and assessment of esophageal injury.

Soldiers with esophageal rupture should be fasted. If wounded soldiers can be evacuated to a specialist hospital within 12 h after the injury, they should be given the priority. If skilled surgeons are available in the early treatment facility and the evacuation time is estimated to be long, repairs should be performed because delayed repair could lead to a high risk of sepsis and high mortality. Conventional procedures of transthoracic esophageal repair are described as follows: rupture of the middle or upper esophagus is most likely in the right chest, therefore, a right thoracotomy should be performed; rupture of the lower esophagus is most likely in the left chest, and thus a left thoracotomy is appropriate. Necrotic and inflammatory tissue should be removed, and accidental damage of the surrounding vital tissues and organs should be avoided. After trimming the edges of the esophageal rupture, a small rupture can be closed with two layers of intermittent sutures and covered by the surrounding pleura or tissue. A larger rupture can be closed with sutures and covered by an intercostal muscle flap or diaphragmatic muscle flap. If the evacuation of the wounded soldiers is delayed and infection has already occurred in the early treatment facility, a mediastinal or chest drainage tube should be placed. If the infection is confined to the upper mediastinum, it can be drained through a cervical incision; if it is in the middle or inferior mediastinum, posterior mediastinal drainage can be performed. For posterior mediastinal drainage, 1–2 corresponding posterior segments of the ribs are removed to expose the posterior mediastinum by retracting the pleura. No damage to the pleura is required in order to avoid infection of the thoracic cavity.

### Diagnosis and early treatment of penetrating thoracoabdominal injury

A combined thoracoabdominal wound refers to the same injury mechanism causing simultaneous damages to the chest, abdominal organs, and diaphragmatic muscle, resulting in an extremely high mortality rate. The incidence of a combined thoracoabdominal wound is not high during peacetime but is higher in wartime. In World War II, the incidence of a combined thoracoabdominal wound was 10–28%, and in the Vietnam and Korean Wars, it was 27–35%. During OEF/OIF, the incidence of a combined thoracoabdominal wound was 4.4% in the British forces [50].

The clinical manifestations of a penetrating combined thoracoabdominal wound are complex and include clinical manifestations of both chest and abdominal injuries. Rapid pulse, lower blood pressure, and other shock symptoms are common. The thoracic injury is mainly manifested as hemopneumothorax. Wounded soldiers have chest pain, dyspnea, and cyanosis. The abdominal injury may be manifested as abdominal pain, abdominal muscle tension, tenderness, and rebound tenderness as well as other peritonitis symptoms. Chest and abdomen X-ray examinations can determine the degree of pneumothorax, hemothorax, and lung collapse, and can observe whether there is free air in the abdominal cavity or abdominal content in the chest. X-ray examinations should be carried out at the bedside for soldiers with severe injuries. The findings of non-coagulating blood or air by thoracentesis indicate a blood vessel injury in the chest or lungs. Abdominocentesis or peritoneal lavage is helpful for the diagnosis of intraabdominal organ injuries [50–53].

The treatment principles for a combined thoracoabdominal wound include adequate respiratory support, maintenance of the water-acid-base balance, infection control, and early surgical treatment. Damage control resuscitation strategies can be used when necessary. Penetrating thoracoabdominal injuries require a laparotomy in most cases, but the decision of whether to open the chest is based on the intraoperative findings. In general, thoracic injury should be treated with closed thoracic drainage. However, if there is progressive hemothorax or continuous massive air leakage, chest exploration should be performed. The procedure sequence between laparotomy and thoracotomy should be determined by the wounded soldiers’ condition. If necessary, two groups of surgeons can perform surgery at the same time. However, rapid hemostasis is the focus of surgical treatment and a thoracoabdominal combined incision should be avoided [50, 52].

### Early treatment of flail chest

The main treatment of the early stage of flail chest is to control and relieve pain. Based on emergency treatment, treating flail chest with towel clip traction is recommended. With advances in treatment, mandatory mechanical ventilation is generally not recommended for fixation of the flail chest wall if the soldier has no signs of respiratory failure in an early treatment facility. When wounded soldiers have signs of respiratory failure, mechanical ventilation can be used, but early weaning of the ventilation should be considered. Positive end-expiratory pressure or continuous positive pressure ventilation are generally recommended [25, 54]. In terms of soldiers with severe flail chest, surgical fixation of the affected ribs is recommended to facilitate early weaning of the ventilation. The affected ribs can be fixated during thoracic surgery when it is required for other reasons. There is no clinical evidence to support which type of fixation is more advantageous. In vitro experiments have shown that the mechanical effects of plate fixation are better than those of intramedullary screw fixation and wire fixation [25, 55].

### Behind armor blunt trauma

In modern warfare, the weapons are more effective than those in previous wars. Even with significant protection from body armor, some mechanical energy can still be transferred to the body. If the bullet fails to penetrate the body armor or the helmet, the mechanism of the body injury changes to behind armor blunt trauma (BABT) [11, 56].

The role of all types of body armor and helmets is to prevent bullets from penetrating the body and to absorb the mechanical energy of the bullet. The severity of the injury depends on the speed of the bullet, the effectiveness of the body armor, and the body contact site and area. Deformation of the body wall and the conduction of energy to internal organs can cause severe injuries and even death. BABT is a common consequence of blunt injury in the battlefield during OIF and OEF. In this case, a systematic assessment should be conducted based on the ATLS. Soldiers with the aforementioned massive thoracic hemorrhage and heart injury should be treated in early treatment facilities [11, 56].

### Consensus 10

Resuscitative emergency thoracotomy should be recommended for wounded soldiers with cardiac arrest and impending cardiac arrest. The left-sided incision or clamshell approach is usually used to open the chest. Then, the pleura and pericardium are opened, the injured aorta is clamped, and intrathoracic CPR is performed. If the heart resuscitation is successful, emergency procedures should be immediately prepared, and the wounded soldier should be transferred to the operating room for further surgical treatment (Class B/Category IIa).

### Consensus 11

Damage control thoracotomy should be performed in soldiers with a progressively massive hemorrhage, severe heart contusions, and severe tracheal and bronchial injuries. The anterolateral left thoracotomy approach is generally used as an initial incision. If necessary, it can be extended to become the clamshell approach. Chest injuries should be treated on a case-by-case basis (Class B/Category IIa).

### Consensus 12

Damage control thoracotomy should be performed for severe pulmonary lacerations in which thoracic closed drainage cannot relieve dyspnea or leads to continuous hemorrhage in the thorax. The surgical methods can be used according to different injury conditions, including repair, lobectomy, segmentectomy, unilateral lung resection, and hilar torsion (Class B/Category IIb).

### Consensus 13

For soldiers with a large rupture of the trachea and bronchus, if tracheotomy and closed thoracic drainage are not able to alleviate the dyspnea, surgical repair should be performed in early treatment facilities. However, if skilled thoracic surgeons are not available, wounded soldiers should be given priority to be evacuated with tracheal intubation and ventilatory support (Class C/Category IIb).

### Consensus 14

For soldiers with penetrating and blunt heart injuries, early diagnosis is required and surgical repair may provide a certain chance of survival. A history of injury in the precordial area, symptoms of tachycardia, signs of enlarged heart shadows on the chest radiograph, and ST-segment elevation in the ECG indicate the possibility of cardiac injury. Ultrasound examination of pericardial trauma can confirm the diagnosis of penetrating cardiac injury, and close monitoring of troponin levels as well as ultrasound should be helpful in improving the diagnostic success rate of blunt heart injury (Class B/Category IIb).

### Consensus 15

In the event of progressive chest hemorrhage caused by penetrating trauma of the thoracic great vessels, thoracotomy should be performed urgently to repair damaged blood vessels or vascular bypass surgery should be performed. Otherwise, wounded soldiers should be quickly evacuated for specialist treatment under close monitoring (Class C/Category IIb).

### Consensus 16

Penetrating injury near the esophagus can cause severe posterior sternal and epigastric pain, dyspnea, and cyanosis, as well as mediastinal and subcutaneous emphysema. The possibility of esophageal injury should be highly considered for wounded soldiers with these symptoms, and mediastinal or subcutaneous emphysema from the chest radiograph can assist in the diagnosis. These soldiers should be given priority for evacuation. If the estimated evacuation time is too long, the repair surgery should be performed in battlefield hospitals (Class C/Category IIb).

### Consensus 17

A combined thoracoabdominal wound is complex and has high mortality. For wounded soldiers who have a definite abdominal organ injury, abdominal damage control surgery should be performed, and the results of closed thoracic drainage can be a reference to determine whether open chest surgery is necessary (Class B/Category IIa).

### Consensus 18

In early treatment facilities, for soldiers with flail chest who have signs of respiratory failure, mechanical ventilation should be used with positive end-expiratory pressure or continuous positive pressure. For soldiers with severe flail chest, surgical fixation of the ribs is recommended to facilitate early weaning of the mechanical ventilation, and the ribs can be fixated during thoracic surgery when it is required for other reasons (Class B/Category IIa).

### Consensus 19

With the increase in the killing effect of weapons, the incidence of BABT is increasing and may lead to serious chest injuries. Corresponding measures should be taken in the early treatment facilities according to different injury conditions (Class B/Category IIa).

## Abbreviations

ATLS: Advanced Trauma Life Support
BABT: Behind armor blunt trauma
CC-CPR: Closed chest cardiopulmonary resuscitation
C-TECC: Committee for Tactical Emergency Casualty Care
DCT: Damage control thoracotomy
ECG: Electrocardiography
OEF: Operation Enduring Freedom
OIF: Operation Iraqi Freedom
PLA: Chinese People’s Liberation Army
